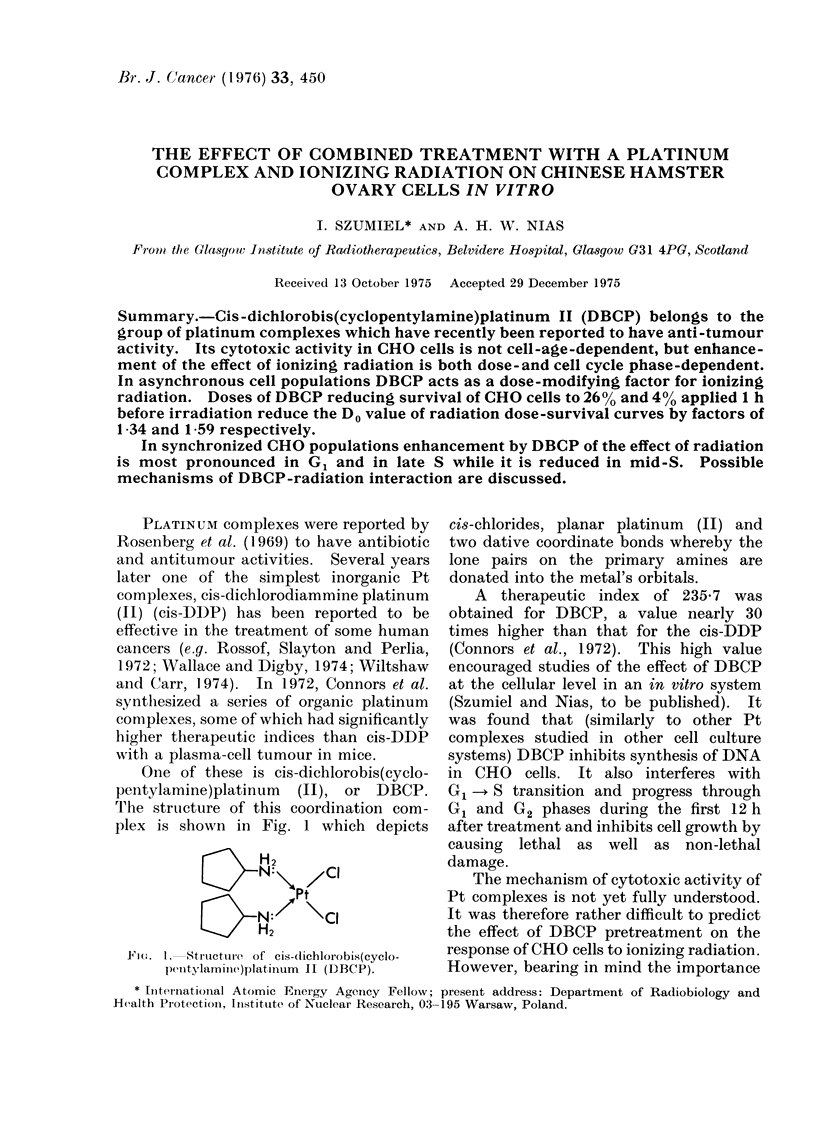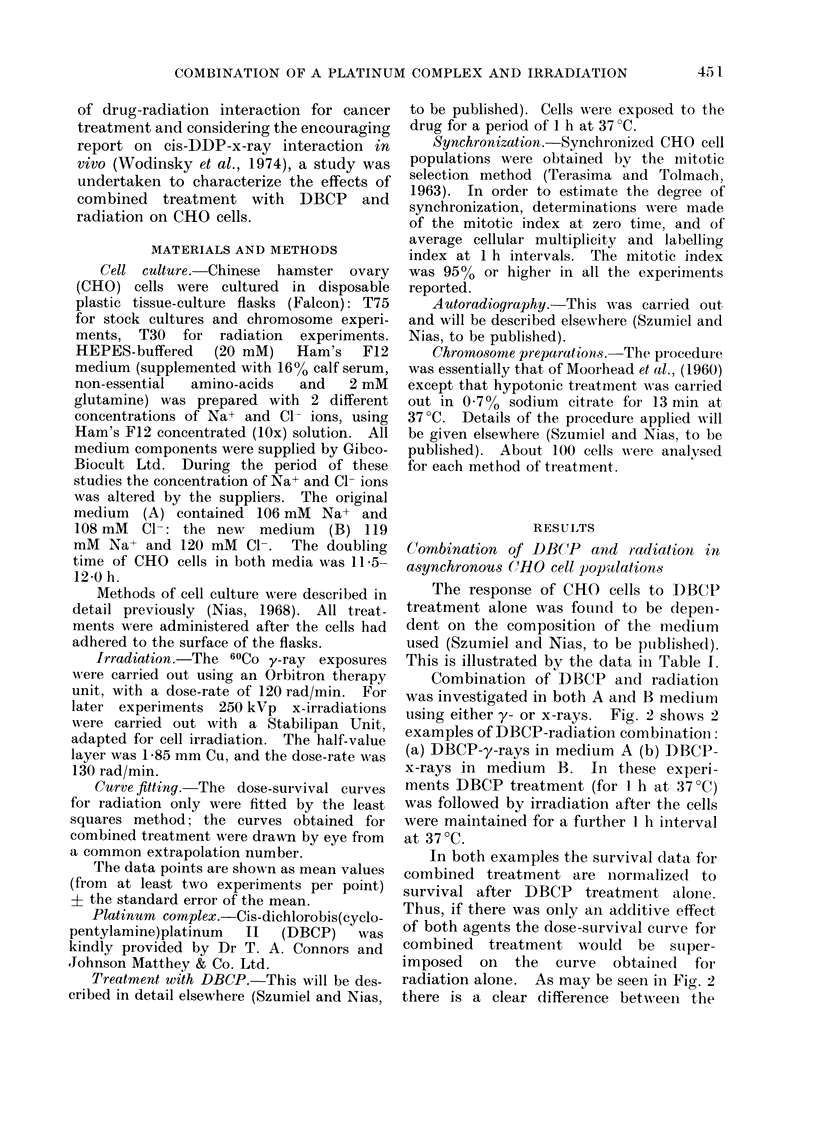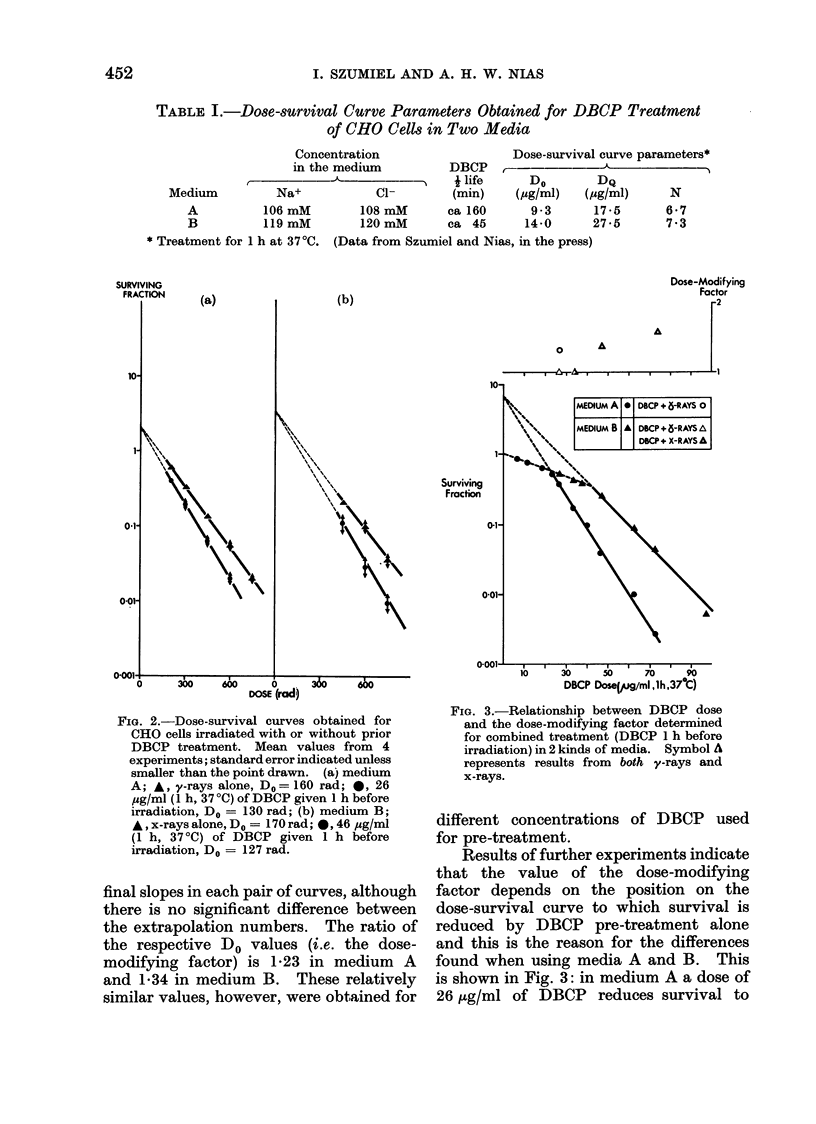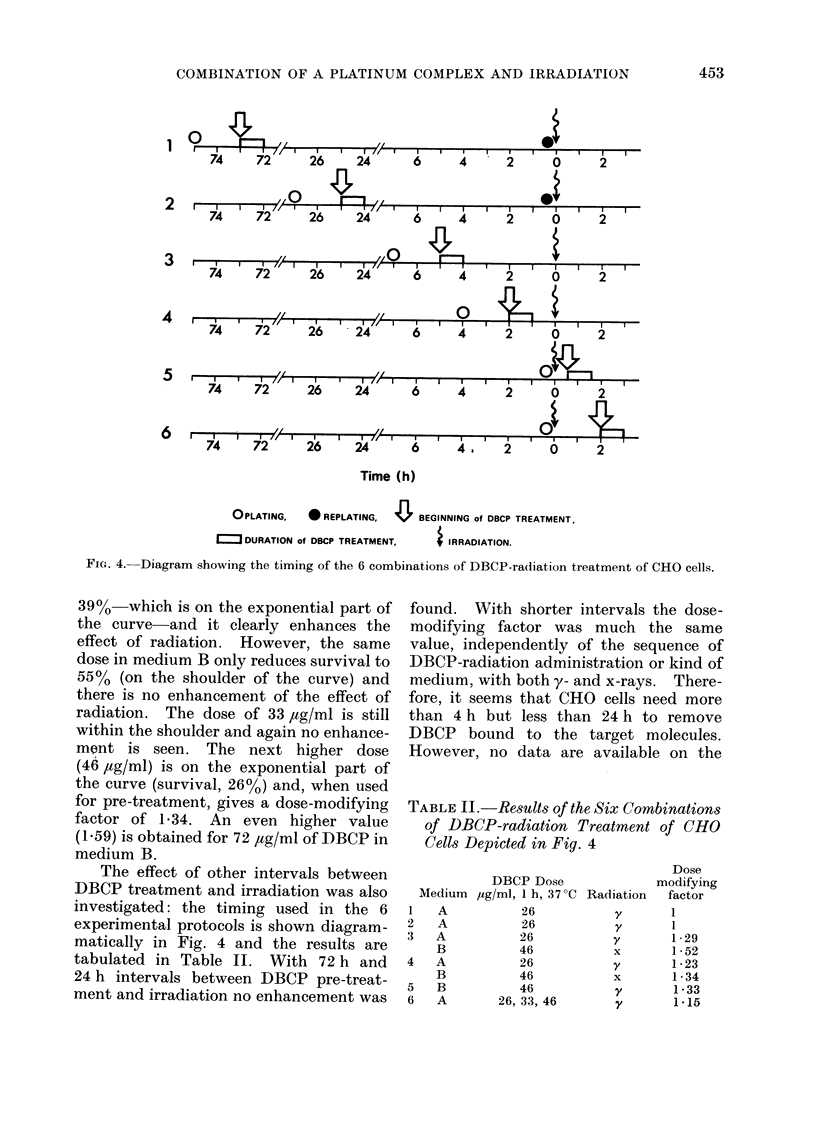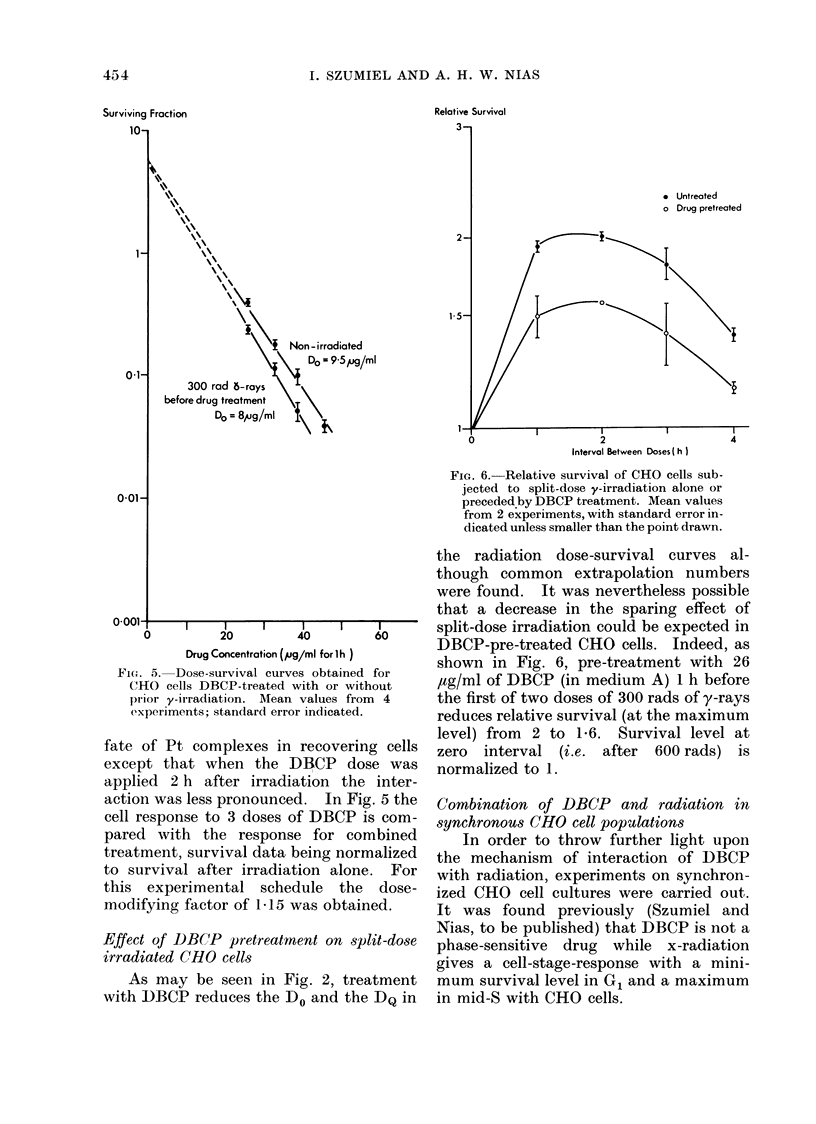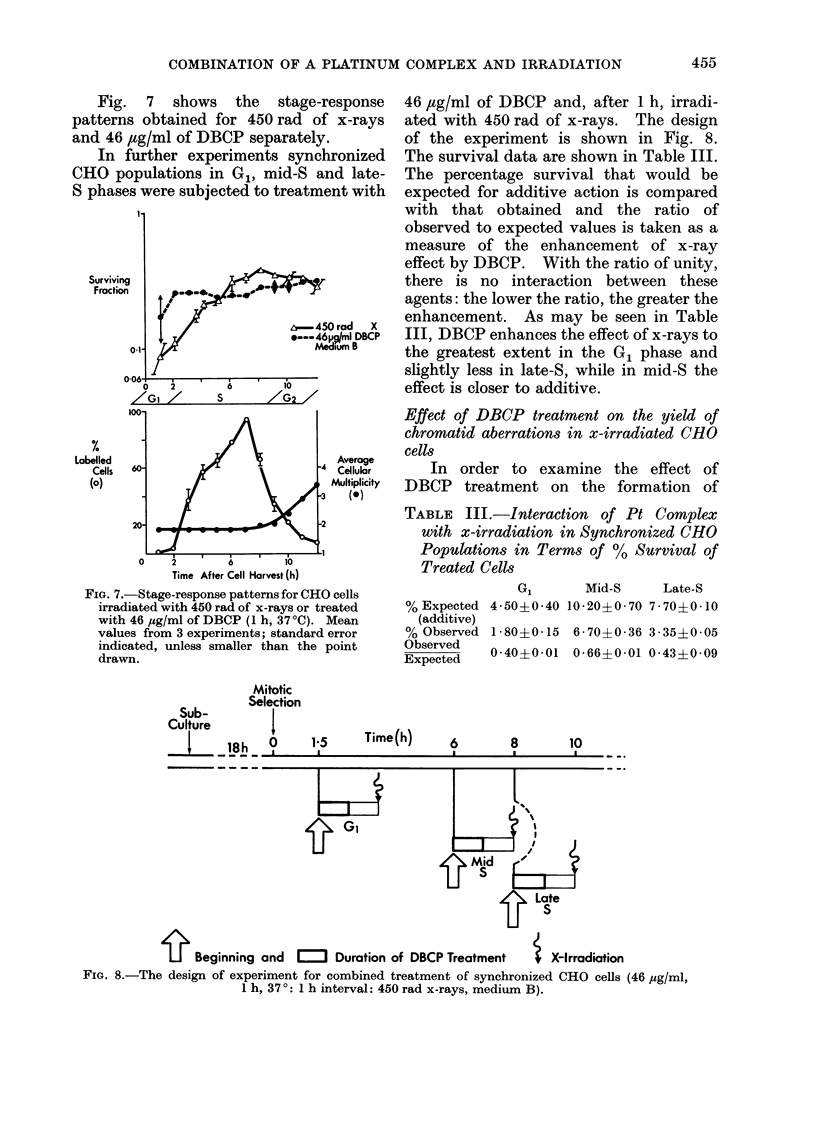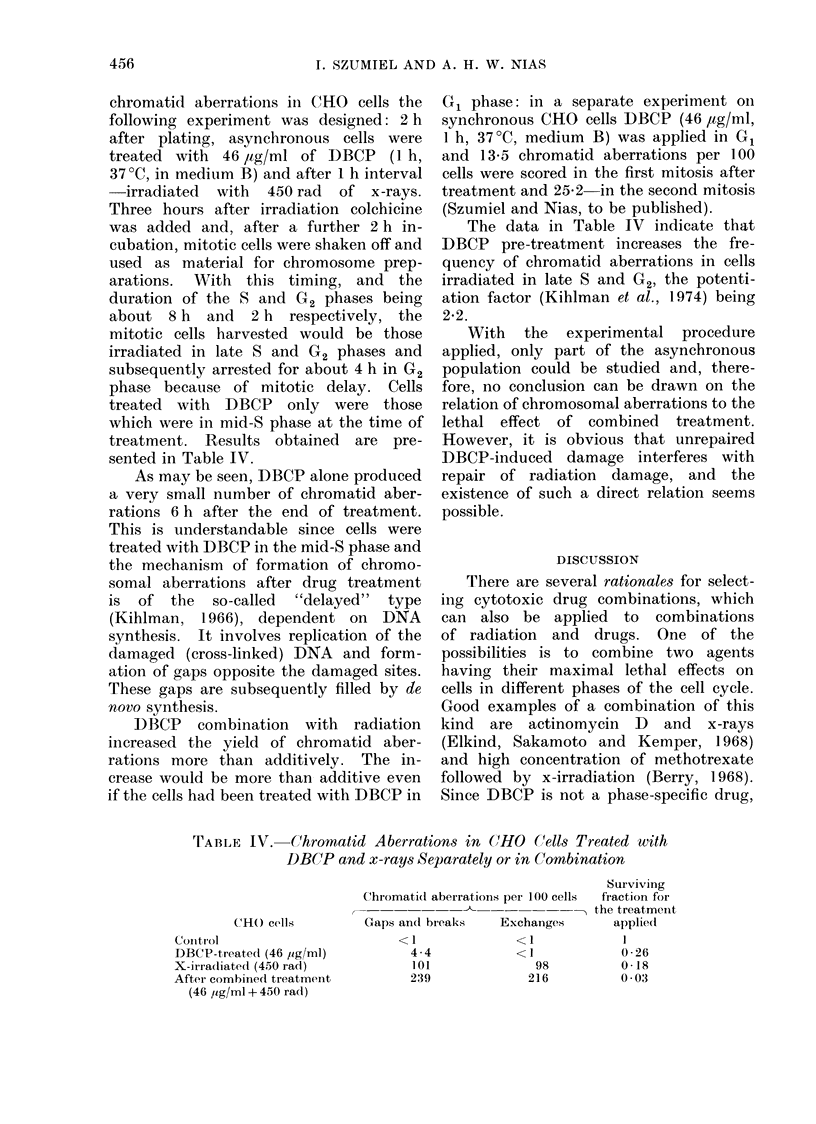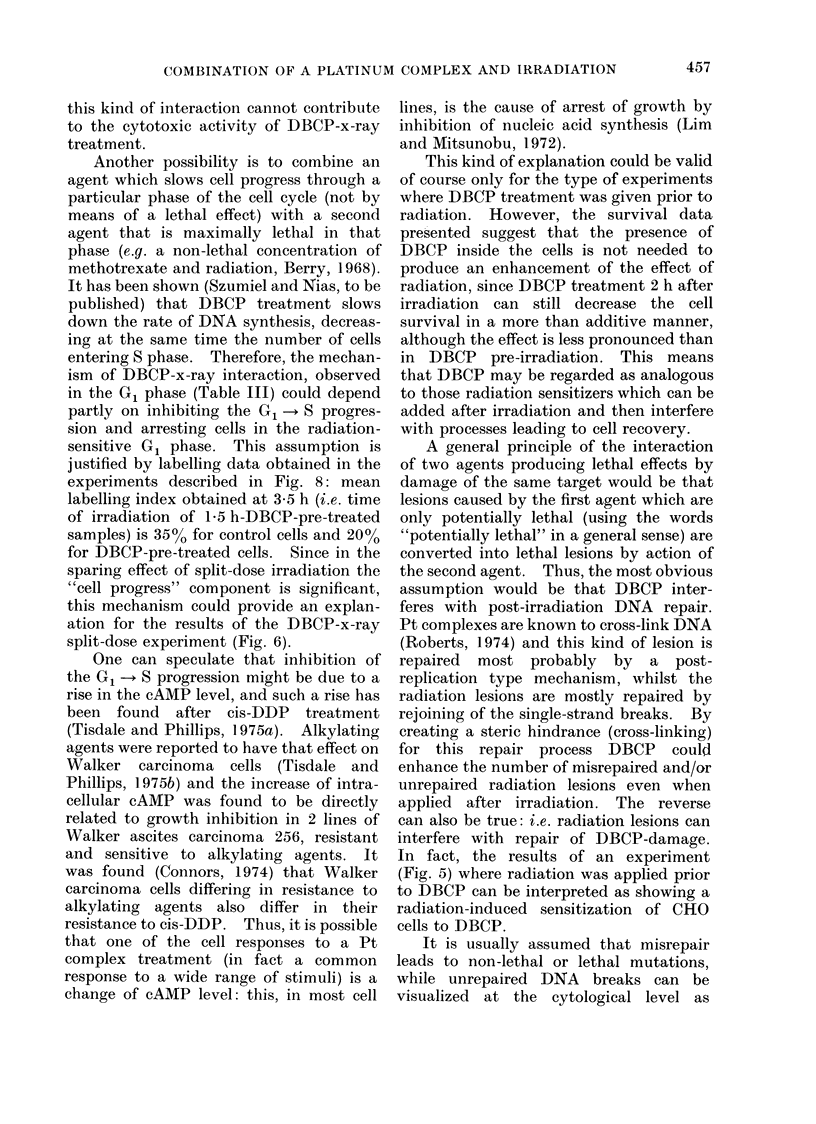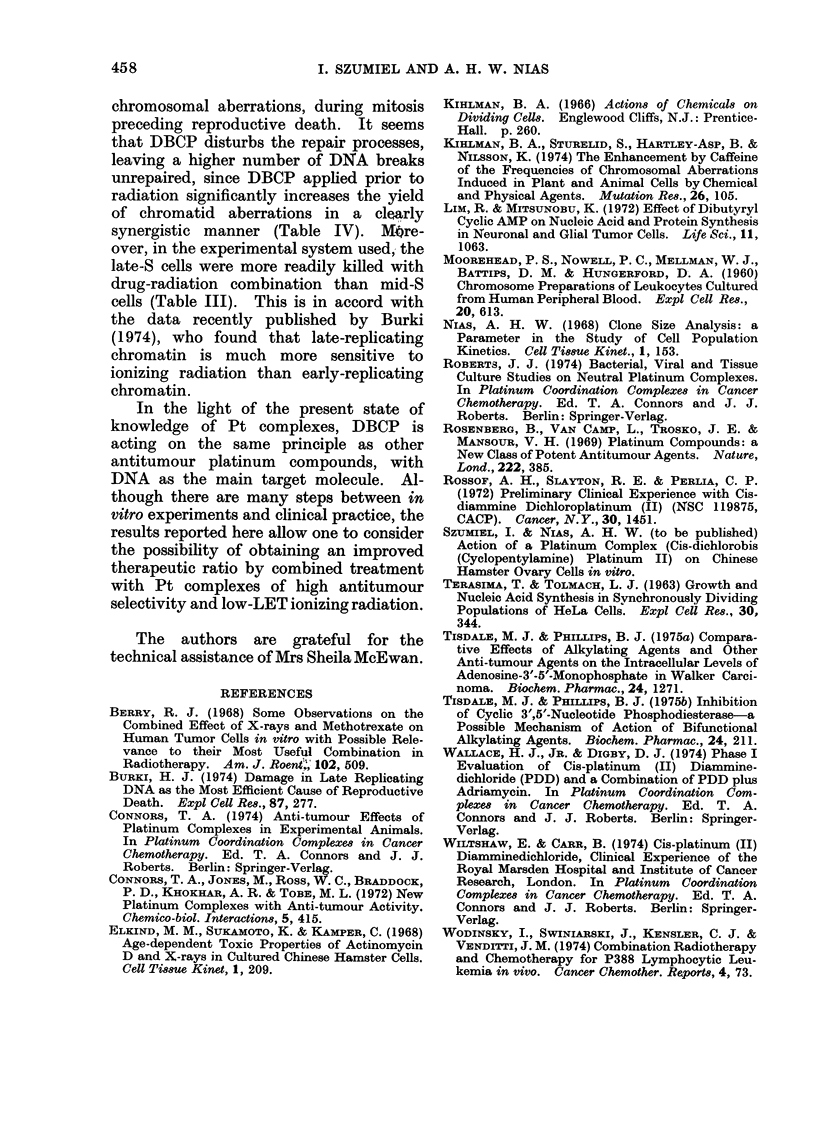# The effect of combined treatment with a platinum complex and ionizing radiation on chinese hamster ovary cells in vitro.

**DOI:** 10.1038/bjc.1976.70

**Published:** 1976-04

**Authors:** I. Szumiel, A. H. Nias

## Abstract

Cis-dichlorobis(cyclopentylamine)platinum II (DBCP) belongs to the group of platinum complexes which have recently been reported to have anti-tumour activity. Its cytotoxic activity in CHO cells is not cell-age-dependent, but enhancement of the effect of ionizing radiation is both dose-and cell cycle phase-dependent. In asynchronous cell populations DBCP acts as a dose-modifying factor for ionizing radiation. Doses of DBCP reducing survival of CHO cells to 26% and 4% applied 1 h before irradiation reduce the Do value of radiation dose-survival curves by factors of 1-34 and 1-59 respectively. In synchronized CHO populations enhancement by DBCP of the effect of radiation is most pronounced in G1 and in late S while it is reduced in mid-S. Possible mechanisms of DBCP-radiation interaction are discussed.


*Br. J. Cancer* (1976) **33**, 450

### THE EFFECT OF COMBINED TREATMENT WITH A PLATINUM COMPLEX AND IONIZING RADIATION ON CHINESE HAMSTER OVARY CELLS *IN VITRO*

I. SZUMIEL* AND A. H. W. NIAS

*From the Glasgow Institute of Radiotherapeutics, Belvidere Hospital, Glasgow G31 4PG, Scotland*

Received 13 October 1975 Accepted 29 December 1975

**Summary.—Cis-dichlorobis(cyclopentylamine)platinum II (DBCP) belongs to the group of platinum complexes which have recently been reported to have anti-tumour activity. Its cytotoxic activity in CHO cells is not cell-age-dependent, but enhancement of the effect of ionizing radiation is both dose-and cell cycle phase-dependent. In asynchronous cell populations DBCP acts as a dose-modifying factor for ionizing radiation. Doses of DBCP reducing survival of CHO cells to 26% and 4% applied 1 h before irradiation reduce the $D_0$ value of radiation dose-survival curves by factors of 1·34 and 1·59 respectively.**

**In synchronized CHO populations enhancement by DBCP of the effect of radiation is most pronounced in $G_1$ and in late S while it is reduced in mid-S. Possible mechanisms of DBCP-radiation interaction are discussed.**

PLATINUM complexes were reported by Rosenberg *et al.* (1969) to have antibiotic and antitumour activities. Several years later one of the simplest inorganic Pt complexes, cis-dichlorodiammine platinum (II) (cis-DDP) has been reported to be effective in the treatment of some human cancers (*e.g.* Rossof, Slayton and Perlia, 1972; Wallace and Digby, 1974; Wiltshaw and Carr, 1974). In 1972, Connors *et al.* synthesized a series of organic platinum complexes, some of which had significantly higher therapeutic indices than cis-DDP with a plasma-cell tumour in mice.

One of these is cis-dichlorobis(cyclopentylamine)platinum (II), or DBCP. The structure of this coordination complex is shown in Fig. 1 which depicts

FIG. 1.—Structure of cis-dichlorobis(cyclopentylamine)platinum II (DBCP).

*cis*-chlorides, planar platinum (II) and two dative coordinate bonds whereby the lone pairs on the primary amines are donated into the metal's orbitals.

A therapeutic index of 235·7 was obtained for DBCP, a value nearly 30 times higher than that for the cis-DDP (Connors *et al.*, 1972). This high value encouraged studies of the effect of DBCP at the cellular level in an *in vitro* system (Szumiel and Nias, to be published). It was found that (similarly to other Pt complexes studied in other cell culture systems) DBCP inhibits synthesis of DNA in CHO cells. It also interferes with $G_1 \rightarrow S$ transition and progress through $G_1$ and $G_2$ phases during the first 12 h after treatment and inhibits cell growth by causing lethal as well as non-lethal damage.

The mechanism of cytotoxic activity of Pt complexes is not yet fully understood. It was therefore rather difficult to predict the effect of DBCP pretreatment on the response of CHO cells to ionizing radiation. However, bearing in mind the importance

* International Atomic Energy Agency Fellow; present address: Department of Radiobiology and Health Protection, Institute of Nuclear Research, 03–195 Warsaw, Poland.

of drug-radiation interaction for cancer treatment and considering the encouraging report on cis-DDP-x-ray interaction *in vivo* (Wodinsky *et al.*, 1974), a study was undertaken to characterize the effects of combined treatment with DBCP and radiation on CHO cells.

### MATERIALS AND METHODS

*Cell culture.*—Chinese hamster ovary (CHO) cells were cultured in disposable plastic tissue-culture flasks (Falcon): T75 for stock cultures and chromosome experiments, T30 for radiation experiments. HEPES-buffered (20 mM) Ham's F12 medium (supplemented with 16% calf serum, non-essential amino-acids and 2 mM glutamine) was prepared with 2 different concentrations of $Na^+$ and $Cl^-$ ions, using Ham's F12 concentrated (10x) solution. All medium components were supplied by Gibco-Biocult Ltd. During the period of these studies the concentration of $Na^+$ and $Cl^-$ ions was altered by the suppliers. The original medium (A) contained 106 mM $Na^+$ and 108 mM $Cl^-$: the new medium (B) 119 mM $Na^+$ and 120 mM $Cl^-$. The doubling time of CHO cells in both media was 11·5–12·0 h.

Methods of cell culture were described in detail previously (Nias, 1968). All treatments were administered after the cells had adhered to the surface of the flasks.

*Irradiation.*—The $^{60}Co$ $\gamma$-ray exposures were carried out using an Orbitron therapy unit, with a dose-rate of 120 rad/min. For later experiments 250 kVp x-irradiations were carried out with a Stabilipan Unit, adapted for cell irradiation. The half-value layer was 1·85 mm Cu, and the dose-rate was 130 rad/min.

*Curve fitting.*—The dose-survival curves for radiation only were fitted by the least squares method; the curves obtained for combined treatment were drawn by eye from a common extrapolation number.

The data points are shown as mean values (from at least two experiments per point) $\pm$ the standard error of the mean.

*Platinum complex.*—Cis-dichlorobis(cyclopentylamine)platinum II (DBCP) was kindly provided by Dr T. A. Connors and Johnson Matthey & Co. Ltd.

*Treatment with DBCP.*—This will be described in detail elsewhere (Szumiel and Nias, to be published). Cells were exposed to the drug for a period of 1 h at 37 °C.

*Synchronization.*—Synchronized CHO cell populations were obtained by the mitotic selection method (Terasima and Tolmach, 1963). In order to estimate the degree of synchronization, determinations were made of the mitotic index at zero time, and of average cellular multiplicity and labelling index at 1 h intervals. The mitotic index was 95% or higher in all the experiments reported.

*Autoradiography.*—This was carried out and will be described elsewhere (Szumiel and Nias, to be published).

*Chromosome preparations.*—The procedure was essentially that of Moorhead *et al.*, (1960) except that hypotonic treatment was carried out in 0·7% sodium citrate for 13 min at 37 °C. Details of the procedure applied will be given elsewhere (Szumiel and Nias, to be published). About 100 cells were analysed for each method of treatment.

### RESULTS

### *Combination of DBCP and radiation in asynchronous CHO cell populations*

The response of CHO cells to DBCP treatment alone was found to be dependent on the composition of the medium used (Szumiel and Nias, to be published). This is illustrated by the data in Table I.

Combination of DBCP and radiation was investigated in both A and B medium using either $\gamma$- or x-rays. Fig. 2 shows 2 examples of DBCP-radiation combination: (a) DBCP-$\gamma$-rays in medium A (b) DBCP-x-rays in medium B. In these experiments DBCP treatment (for 1 h at 37 °C) was followed by irradiation after the cells were maintained for a further 1 h interval at 37 °C.

In both examples the survival data for combined treatment are normalized to survival after DBCP treatment alone. Thus, if there was only an additive effect of both agents the dose-survival curve for combined treatment would be superimposed on the curve obtained for radiation alone. As may be seen in Fig. 2 there is a clear difference between the

TABLE I.—*Dose-survival Curve Parameters Obtained for DBCP Treatment of CHO Cells in Two Media*

| Medium | Concentration in the medium | | DBCP ½ life (min) | Dose-survival curve parameters* | | |
|---|---|---|---|---|---|---|
| | $Na^+$ | $Cl^-$ | | $D_0$ (μg/ml) | $D_Q$ (μg/ml) | N |
| A | 106 mM | 108 mM | ca 160 | 9·3 | 17·5 | 6·7 |
| B | 119 mM | 120 mM | ca 45 | 14·0 | 27·5 | 7·3 |

* Treatment for 1 h at 37 °C. (Data from Szumiel and Nias, in the press)

FIG. 2.—Dose-survival curves obtained for CHO cells irradiated with or without prior DBCP treatment. Mean values from 4 experiments; standard error indicated unless smaller than the point drawn. (a) medium A; ▲, γ-rays alone, $D_0 = 160$ rad; ●, 26 μg/ml (1 h, 37 °C) of DBCP given 1 h before irradiation, $D_0 = 130$ rad; (b) medium B; ▲, x-rays alone, $D_0 = 170$ rad; ●, 46 μg/ml (1 h, 37 °C) of DBCP given 1 h before irradiation, $D_0 = 127$ rad.

FIG. 3.—Relationship between DBCP dose and the dose-modifying factor determined for combined treatment (DBCP 1 h before irradiation) in 2 kinds of media. Symbol ▲ represents results from *both* γ-rays and x-rays.

final slopes in each pair of curves, although there is no significant difference between the extrapolation numbers. The ratio of the respective $D_0$ values (*i.e.* the dose-modifying factor) is 1·23 in medium A and 1·34 in medium B. These relatively similar values, however, were obtained for different concentrations of DBCP used for pre-treatment.

Results of further experiments indicate that the value of the dose-modifying factor depends on the position on the dose-survival curve to which survival is reduced by DBCP pre-treatment alone and this is the reason for the differences found when using media A and B. This is shown in Fig. 3: in medium A a dose of 26 μg/ml of DBCP reduces survival to

FIG. 4.—Diagram showing the timing of the 6 combinations of DBCP-radiation treatment of CHO cells.

39%—which is on the exponential part of the curve—and it clearly enhances the effect of radiation. However, the same dose in medium B only reduces survival to 55% (on the shoulder of the curve) and there is no enhancement of the effect of radiation. The dose of 33 μg/ml is still within the shoulder and again no enhancement is seen. The next higher dose (46 μg/ml) is on the exponential part of the curve (survival, 26%) and, when used for pre-treatment, gives a dose-modifying factor of 1·34. An even higher value (1·59) is obtained for 72 μg/ml of DBCP in medium B.

The effect of other intervals between DBCP treatment and irradiation was also investigated: the timing used in the 6 experimental protocols is shown diagrammatically in Fig. 4 and the results are tabulated in Table II. With 72 h and 24 h intervals between DBCP pre-treatment and irradiation no enhancement was found. With shorter intervals the dose-modifying factor was much the same value, independently of the sequence of DBCP-radiation administration or kind of medium, with both γ- and x-rays. Therefore, it seems that CHO cells need more than 4 h but less than 24 h to remove DBCP bound to the target molecules. However, no data are available on the

TABLE II.—*Results of the Six Combinations of DBCP-radiation Treatment of CHO Cells Depicted in Fig.* 4

| | Medium | DBCP Dose μg/ml, 1 h, 37 °C | Radiation | Dose modifying factor |
|---|---|---|---|---|
| 1 | A | 26 | γ | 1 |
| 2 | A | 26 | γ | 1 |
| 3 | A | 26 | γ | 1·29 |
| | B | 46 | x | 1·52 |
| 4 | A | 26 | γ | 1·23 |
| | B | 46 | x | 1·34 |
| 5 | B | 46 | γ | 1·33 |
| 6 | A | 26, 33, 46 | γ | 1·15 |

FIG. 5.—Dose-survival curves obtained for CHO cells DBCP-treated with or without prior $\gamma$-irradiation. Mean values from 4 experiments; standard error indicated.

FIG. 6.—Relative survival of CHO cells subjected to split-dose $\gamma$-irradiation alone or preceded by DBCP treatment. Mean values from 2 experiments, with standard error indicated unless smaller than the point drawn.

fate of Pt complexes in recovering cells except that when the DBCP dose was applied 2 h after irradiation the interaction was less pronounced. In Fig. 5 the cell response to 3 doses of DBCP is compared with the response for combined treatment, survival data being normalized to survival after irradiation alone. For this experimental schedule the dose-modifying factor of 1·15 was obtained.

*Effect of DBCP pretreatment on split-dose irradiated CHO cells*

As may be seen in Fig. 2, treatment with DBCP reduces the $D_0$ and the $D_Q$ in the radiation dose-survival curves although common extrapolation numbers were found. It was nevertheless possible that a decrease in the sparing effect of split-dose irradiation could be expected in DBCP-pre-treated CHO cells. Indeed, as shown in Fig. 6, pre-treatment with 26 μg/ml of DBCP (in medium A) 1 h before the first of two doses of 300 rads of $\gamma$-rays reduces relative survival (at the maximum level) from 2 to 1·6. Survival level at zero interval (*i.e.* after 600 rads) is normalized to 1.

*Combination of DBCP and radiation in synchronous CHO cell populations*

In order to throw further light upon the mechanism of interaction of DBCP with radiation, experiments on synchronized CHO cell cultures were carried out. It was found previously (Szumiel and Nias, to be published) that DBCP is not a phase-sensitive drug while x-radiation gives a cell-stage-response with a minimum survival level in $G_1$ and a maximum in mid-S with CHO cells.

Fig. 7 shows the stage-response patterns obtained for 450 rad of x-rays and 46 μg/ml of DBCP separately.

In further experiments synchronized CHO populations in $G_1$, mid-S and late-S phases were subjected to treatment with 46 μg/ml of DBCP and, after 1 h, irradiated with 450 rad of x-rays. The design of the experiment is shown in Fig. 8. The survival data are shown in Table III. The percentage survival that would be expected for additive action is compared with that obtained and the ratio of observed to expected values is taken as a measure of the enhancement of x-ray effect by DBCP. With the ratio of unity, there is no interaction between these agents: the lower the ratio, the greater the enhancement. As may be seen in Table III, DBCP enhances the effect of x-rays to the greatest extent in the $G_1$ phase and slightly less in late-S, while in mid-S the effect is closer to additive.

FIG. 7.—Stage-response patterns for CHO cells irradiated with 450 rad of x-rays or treated with 46 μg/ml of DBCP (1 h, 37 °C). Mean values from 3 experiments; standard error indicated, unless smaller than the point drawn.

*Effect of DBCP treatment on the yield of chromatid aberrations in x-irradiated CHO cells*

In order to examine the effect of DBCP treatment on the formation of

TABLE III.—*Interaction of Pt Complex with x-irradiation in Synchronized CHO Populations in Terms of % Survival of Treated Cells*

| | $G_1$ | Mid-S | Late-S |
|---|---|---|---|
| % Expected (additive) | 4·50±0·40 | 10·20±0·70 | 7·70±0·10 |
| % Observed | 1·80±0·15 | 6·70±0·36 | 3·35±0·05 |
| $\frac{\text{Observed}}{\text{Expected}}$ | 0·40±0·01 | 0·66±0·01 | 0·43±0·09 |

FIG. 8.—The design of experiment for combined treatment of synchronized CHO cells (46 μg/ml, 1 h, 37°: 1 h interval: 450 rad x-rays, medium B).

chromatid aberrations in CHO cells the following experiment was designed: 2 h after plating, asynchronous cells were treated with 46 $\mu$g/ml of DBCP (1 h, 37 °C, in medium B) and after 1 h interval —irradiated with 450 rad of x-rays. Three hours after irradiation colchicine was added and, after a further 2 h incubation, mitotic cells were shaken off and used as material for chromosome preparations. With this timing, and the duration of the S and $G_2$ phases being about 8 h and 2 h respectively, the mitotic cells harvested would be those irradiated in late S and $G_2$ phases and subsequently arrested for about 4 h in $G_2$ phase because of mitotic delay. Cells treated with DBCP only were those which were in mid-S phase at the time of treatment. Results obtained are presented in Table IV.

As may be seen, DBCP alone produced a very small number of chromatid aberrations 6 h after the end of treatment. This is understandable since cells were treated with DBCP in the mid-S phase and the mechanism of formation of chromosomal aberrations after drug treatment is of the so-called "delayed" type (Kihlman, 1966), dependent on DNA synthesis. It involves replication of the damaged (cross-linked) DNA and formation of gaps opposite the damaged sites. These gaps are subsequently filled by *de novo* synthesis.

DBCP combination with radiation increased the yield of chromatid aberrations more than additively. The increase would be more than additive even if the cells had been treated with DBCP in $G_1$ phase: in a separate experiment on synchronous CHO cells DBCP (46 $\mu$g/ml, 1 h, 37 °C, medium B) was applied in $G_1$ and 13·5 chromatid aberrations per 100 cells were scored in the first mitosis after treatment and 25·2—in the second mitosis (Szumiel and Nias, to be published).

The data in Table IV indicate that DBCP pre-treatment increases the frequency of chromatid aberrations in cells irradiated in late S and $G_2$, the potentiation factor (Kihlman *et al.*, 1974) being 2·2.

With the experimental procedure applied, only part of the asynchronous population could be studied and, therefore, no conclusion can be drawn on the relation of chromosomal aberrations to the lethal effect of combined treatment. However, it is obvious that unrepaired DBCP-induced damage interferes with repair of radiation damage, and the existence of such a direct relation seems possible.

### DISCUSSION

There are several *rationales* for selecting cytotoxic drug combinations, which can also be applied to combinations of radiation and drugs. One of the possibilities is to combine two agents having their maximal lethal effects on cells in different phases of the cell cycle. Good examples of a combination of this kind are actinomycin D and x-rays (Elkind, Sakamoto and Kemper, 1968) and high concentration of methotrexate followed by x-irradiation (Berry, 1968). Since DBCP is not a phase-specific drug,

TABLE IV.—*Chromatid Aberrations in CHO Cells Treated with DBCP and x-rays Separately or in Combination*

| CHO cells | Chromatid aberrations per 100 cells: Gaps and breaks | Chromatid aberrations per 100 cells: Exchanges | Surviving fraction for the treatment applied |
|---|---|---|---|
| Control | <1 | <1 | 1 |
| DBCP-treated (46 $\mu$g/ml) | 4·4 | <1 | 0·26 |
| X-irradiated (450 rad) | 101 | 98 | 0·18 |
| After combined treatment (46 $\mu$g/ml + 450 rad) | 239 | 216 | 0·03 |

this kind of interaction cannot contribute to the cytotoxic activity of DBCP-x-ray treatment.

Another possibility is to combine an agent which slows cell progress through a particular phase of the cell cycle (not by means of a lethal effect) with a second agent that is maximally lethal in that phase (*e.g.* a non-lethal concentration of methotrexate and radiation, Berry, 1968). It has been shown (Szumiel and Nias, to be published) that DBCP treatment slows down the rate of DNA synthesis, decreasing at the same time the number of cells entering S phase. Therefore, the mechanism of DBCP-x-ray interaction, observed in the $G_1$ phase (Table III) could depend partly on inhibiting the $G_1 \rightarrow S$ progression and arresting cells in the radiation-sensitive $G_1$ phase. This assumption is justified by labelling data obtained in the experiments described in Fig. 8: mean labelling index obtained at 3·5 h (*i.e.* time of irradiation of 1·5 h-DBCP-pre-treated samples) is 35% for control cells and 20% for DBCP-pre-treated cells. Since in the sparing effect of split-dose irradiation the "cell progress" component is significant, this mechanism could provide an explanation for the results of the DBCP-x-ray split-dose experiment (Fig. 6).

One can speculate that inhibition of the $G_1 \rightarrow S$ progression might be due to a rise in the cAMP level, and such a rise has been found after cis-DDP treatment (Tisdale and Phillips, 1975*a*). Alkylating agents were reported to have that effect on Walker carcinoma cells (Tisdale and Phillips, 1975*b*) and the increase of intracellular cAMP was found to be directly related to growth inhibition in 2 lines of Walker ascites carcinoma 256, resistant and sensitive to alkylating agents. It was found (Connors, 1974) that Walker carcinoma cells differing in resistance to alkylating agents also differ in their resistance to cis-DDP. Thus, it is possible that one of the cell responses to a Pt complex treatment (in fact a common response to a wide range of stimuli) is a change of cAMP level: this, in most cell lines, is the cause of arrest of growth by inhibition of nucleic acid synthesis (Lim and Mitsunobu, 1972).

This kind of explanation could be valid of course only for the type of experiments where DBCP treatment was given prior to radiation. However, the survival data presented suggest that the presence of DBCP inside the cells is not needed to produce an enhancement of the effect of radiation, since DBCP treatment 2 h after irradiation can still decrease the cell survival in a more than additive manner, although the effect is less pronounced than in DBCP pre-irradiation. This means that DBCP may be regarded as analogous to those radiation sensitizers which can be added after irradiation and then interfere with processes leading to cell recovery.

A general principle of the interaction of two agents producing lethal effects by damage of the same target would be that lesions caused by the first agent which are only potentially lethal (using the words "potentially lethal" in a general sense) are converted into lethal lesions by action of the second agent. Thus, the most obvious assumption would be that DBCP interferes with post-irradiation DNA repair. Pt complexes are known to cross-link DNA (Roberts, 1974) and this kind of lesion is repaired most probably by a post-replication type mechanism, whilst the radiation lesions are mostly repaired by rejoining of the single-strand breaks. By creating a steric hindrance (cross-linking) for this repair process DBCP could enhance the number of misrepaired and/or unrepaired radiation lesions even when applied after irradiation. The reverse can also be true: *i.e.* radiation lesions can interfere with repair of DBCP-damage. In fact, the results of an experiment (Fig. 5) where radiation was applied prior to DBCP can be interpreted as showing a radiation-induced sensitization of CHO cells to DBCP.

It is usually assumed that misrepair leads to non-lethal or lethal mutations, while unrepaired DNA breaks can be visualized at the cytological level as chromosomal aberrations, during mitosis preceding reproductive death. It seems that DBCP disturbs the repair processes, leaving a higher number of DNA breaks unrepaired, since DBCP applied prior to radiation significantly increases the yield of chromatid aberrations in a clearly synergistic manner (Table IV). Moreover, in the experimental system used, the late-S cells were more readily killed with drug-radiation combination than mid-S cells (Table III). This is in accord with the data recently published by Burki (1974), who found that late-replicating chromatin is much more sensitive to ionizing radiation than early-replicating chromatin.

In the light of the present state of knowledge of Pt complexes, DBCP is acting on the same principle as other antitumour platinum compounds, with DNA as the main target molecule. Although there are many steps between *in vitro* experiments and clinical practice, the results reported here allow one to consider the possibility of obtaining an improved therapeutic ratio by combined treatment with Pt complexes of high antitumour selectivity and low-LET ionizing radiation.

The authors are grateful for the technical assistance of Mrs Sheila McEwan.

### REFERENCES

BERRY, R. J. (1968) Some Observations on the Combined Effect of X-rays and Methotrexate on Human Tumor Cells *in vitro* with Possible Relevance to their Most Useful Combination in Radiotherapy. *Am. J. Roent.*, **102**, 509.

BURKI, H. J. (1974) Damage in Late Replicating DNA as the Most Efficient Cause of Reproductive Death. *Expl Cell Res.*, **87**, 277.

CONNORS, T. A. (1974) Anti-tumour Effects of Platinum Complexes in Experimental Animals. In *Platinum Coordination Complexes in Cancer Chemotherapy*. Ed. T. A. Connors and J. J. Roberts. Berlin: Springer-Verlag.

CONNORS, T. A., JONES, M., ROSS, W. C., BRADDOCK, P. D., KHOKHAR, A. R. & TOBE, M. L. (1972) New Platinum Complexes with Anti-tumour Activity. *Chemico-biol. Interactions*, **5**, 415.

ELKIND, M. M., SUKAMOTO, K. & KAMPER, C. (1968) Age-dependent Toxic Properties of Actinomycin D and X-rays in Cultured Chinese Hamster Cells. *Cell Tissue Kinet*, **1**, 209.

KIHLMAN, B. A. (1966) *Actions of Chemicals on Dividing Cells*. Englewood Cliffs, N.J.: Prentice-Hall. p. 260.

KIHLMAN, B. A., STURELID, S., HARTLEY-ASP, B. & NILSSON, K. (1974) The Enhancement by Caffeine of the Frequencies of Chromosomal Aberrations Induced in Plant and Animal Cells by Chemical and Physical Agents. *Mutation Res.*, **26**, 105.

LIM, R. & MITSUNOBU, K. (1972) Effect of Dibutyryl Cyclic AMP on Nucleic Acid and Protein Synthesis in Neuronal and Glial Tumor Cells. *Life Sci.*, **11**, 1063.

MOOREHEAD, P. S., NOWELL, P. C., MELLMAN, W. J., BATTIPS, D. M. & HUNGERFORD, D. A. (1960) Chromosome Preparations of Leukocytes Cultured from Human Peripheral Blood. *Expl Cell Res.*, **20**, 613.

NIAS, A. H. W. (1968) Clone Size Analysis: a Parameter in the Study of Cell Population Kinetics. *Cell Tissue Kinet.*, **1**, 153.

ROBERTS, J. J. (1974) Bacterial, Viral and Tissue Culture Studies on Neutral Platinum Complexes. In *Platinum Coordination Complexes in Cancer Chemotherapy*. Ed. T. A. Connors and J. J. Roberts. Berlin: Springer-Verlag.

ROSENBERG, B., VAN CAMP, L., TROSKO, J. E. & MANSOUR, V. H. (1969) Platinum Compounds: a New Class of Potent Antitumour Agents. *Nature, Lond.*, **222**, 385.

ROSSOF, A. H., SLAYTON, R. E. & PERLIA, C. P. (1972) Preliminary Clinical Experience with Cis-diammine Dichloroplatinum (II) (NSC 119875, CACP). *Cancer, N.Y.*, **30**, 1451.

SZUMIEL, I. & NIAS, A. H. W. (to be published) Action of a Platinum Complex (Cis-dichlorobis (Cyclopentylamine) Platinum II) on Chinese Hamster Ovary Cells *in vitro*.

TERASIMA, T. & TOLMACH, L. J. (1963) Growth and Nucleic Acid Synthesis in Synchronously Dividing Populations of HeLa Cells. *Expl Cell Res.*, **30**, 344.

TISDALE, M. J. & PHILLIPS, B. J. (1975*a*) Comparative Effects of Alkylating Agents and Other Anti-tumour Agents on the Intracellular Levels of Adenosine-3′-5′-Monophosphate in Walker Carcinoma. *Biochem. Pharmac.*, **24**, 1271.

TISDALE, M. J. & PHILLIPS, B. J. (1975*b*) Inhibition of Cyclic 3′,5′-Nucleotide Phosphodiesterase—a Possible Mechanism of Action of Bifunctional Alkylating Agents. *Biochem. Pharmac.*, **24**, 211.

WALLACE, H. J., JR. & DIGBY, D. J. (1974) Phase I Evaluation of Cis-platinum (II) Diammine-dichloride (PDD) and a Combination of PDD plus Adriamycin. In *Platinum Coordination Complexes in Cancer Chemotherapy*. Ed. T. A. Connors and J. J. Roberts. Berlin: Springer-Verlag.

WILTSHAW, E. & CARR, B. (1974) Cis-platinum (II) Diamminedichloride, Clinical Experience of the Royal Marsden Hospital and Institute of Cancer Research, London. In *Platinum Coordination Complexes in Cancer Chemotherapy*. Ed. T. A. Connors and J. J. Roberts. Berlin: Springer-Verlag.

WODINSKY, I., SWINIARSKI, J., KENSLER, C. J. & VENDITTI, J. M. (1974) Combination Radiotherapy and Chemotherapy for P388 Lymphocytic Leukemia *in vivo*. *Cancer Chemother. Reports*, **4**, 73.